# Assessing Roadside Hybrid Energy Absorbers Using the Example of SafeEnd

**DOI:** 10.3390/ma15051712

**Published:** 2022-02-24

**Authors:** Marcin Budzynski, Kazimierz Jamroz, Lukasz Jelinski, Dawid Bruski, Lukasz Pachocki, Grzegorz Baginski

**Affiliations:** 1 Faculty of Civil and Environmental Engineering, Gdansk University of Technology, 80-233 Gdansk, Poland; kjamroz@pg.edu.pl (K.J.); lukjelin@pg.edu.pl (L.J.); dawid.bruski@pg.edu.pl (D.B.); lukpacho@pg.edu.pl (L.P.); 2 Saferoad Sp. z o.o., 87-800 Wloclawek, Poland; grzegorz.baginski@saferoad.pl

**Keywords:** road safety, road restraint system, crash and numerical tests

## Abstract

A combination of crash cushion and end-terminal, hybrid energy absorbing devices have been in use worldwide for a few years already. They include SafeEnd, a system Poland has recently introduced. Some road authorities have raised concerns as regards the operating conditions of the devices and how they work together with safety barriers. The objective of this research is to clarify the concerns and answer the following questions: (1) Can SafeEnd devices be used as hybrid devices and combine the roles of end-terminal and crash cushion placed before an obstacle? (2) What should be the rules for installing crash cushions at diverging roads and at the start of an off-ramp? The article presents characteristics of SafeEnd devices, defines the doubts raised by road safety auditors, discusses the results of field and numerical tests of the devices and explains the design principles for interchange ramps where crash cushions are required. The study results have helped to answer the research questions: SafeEnd devices fulfil the role of end-terminal and crash cushion, it is possible to make them more visible and principles have been defined for how the devices should be used at road interchanges. Further research should help to define general principles of deploying road restraint systems such as crashworthy terminals, crash cushions or hybrid devices.

## 1. Introduction

Roadsides are critical to road safety management. Roadside hazards represent one of the main causes of fatal accidents [1,2,3]. One of the ways to manage road safety is to use energy absorbers that prevent vehicles from running off the road [4,5].

Energy absorbers that act as crash cushions and end-terminals have been in use for a few years worldwide. The devices are designed to shield road barrier terminals and protect objects in the clear zone. Recently introduced in Poland, the energy absorber SafeEnd (SE) is an example of such devices. Some road authorities, however, have been raising concerns over their use as an energy absorber. Road authorities mainly object to the fact that:

- The SE system is not certified as a crash cushion,
- The SE is connected to barriers at interchange ramps in a way that increases the deflection of safety barriers from road edge; the result is that the barrier may be hit at more than 20 degrees which may increase the risk of barrier penetration and hitting an obstacle.

The article presents the results of research and SE operation analyses. It also looks at the possibilities and principles of using the devices as hybrid energy absorbers. Answers have been formulated to the research question: ‘Can SE devices be used as hybrid devices fulfilling the role of both an end-terminal, barrier terminal and crash cushion placed before an obstacle?’.

The work presented in the article covers an energy absorber which is a hybrid combination of the functions of a crashworthy terminal and crash cushion [6,7]. Crashworthy terminals are road safety devices, also known as energy absorbers, which are designed to reduce the severity of impact of a vehicle crashing into the start or end section of a road safety barrier [8,9]. They are characteristically permanently attached to the safety barrier. Crash cushions are devices designed to reduce the severity of impact of a car hitting a stationary obstacle (e.g., a bridge support) or entering an area at risk. There are two types of crash cushions: redirective and non-redirective [10,11]. A non-redirective cushion is designed to slow and contain an impacting vehicle. Its role is to take over the force whose direction is consistent with or similar to its design axis. It is used in locations where it is highly likely that a vehicle will impact the cushion head-on rather than from a high angle or from the side (e.g., toll booths on motorways). A redirective cushion during a side impact acts similarly to a safety barrier—it reduces the speed and kinetic energy of an impacting vehicle, changes its direction and puts it back in the expected direction (e.g., at the start of off-ramps).

When road restraint systems such as safety barriers with end-terminals or crash cushions are analysed for their applicability, the number one principle is to design roadsides that do not require such devices [12,13,14,15]. Hence the terms ‘safety zone’ or ‘clear zone’ which implies zones clear of obstacles that may lead to serious injury or fatality accidents [16,17,18,19]. This approach fits in with the idea of ‘forgiving roads’ [2,3,20].

The first research on crashworthy terminals and crash cushions dates back to the 1970s. J.G. Viner in his work highlighted the advantages of protecting the start sections of safety barriers and obstacles [21,22,23]. Bender and Zuker presented ways to protect barrier terminals and obstacles using interconnected water-filled cylinders [24]. In subsequent years, improvements were made to the technical parameters of end-terminals and crash cushions. Moreover, crash test standards were introduced for these devices [25,26]. Works carried out in the 1980s looked at the consequences of small vehicles impacting barrier terminals and pointed out that vehicle occupants suffered severe consequences [27]. In the 1980s, crashworthy terminals and crash cushions became widely used especially in the USA with research concentrating on their design and the consequences for car occupants [28,29]. In 1981 NCHRP 230 was developed, a report on procedures for testing road restraint systems [30]. Results of research were presented on the effects of safer barrier terminals on road user safety [31]. Zeeger pointed out that the individual elements of road infrastructure, including crash cushions, should be assessed for the hazard they pose which is a function of the likelihood that the particular element will be linked to an accident and its consequences [32]. Assessments of the hazards involved in barrier terminal impacts suggested that solutions are needed to mitigate the consequences of such an accident [33]. Solutions were sought to both improving safety and keeping maintenance costs low. As an example, analysis results were presented of the operation of a crashworthy terminal at the end of a concrete barrier [34] and a crash cushion with rubber elements [35]. Devices which until then were mostly used in the USA were picked up by other countries, e.g., in the UK which in 1987 installed the country’s first crash cushions on the Birmingham ringroad at so-called black spots (high accident risk spots) [36]. The results of research by Green et al. prove that safety barriers and crash cushions are also obstacles and should only be installed where accident consequences could be more severe in their absence [37]. Other research shows that as a priority action all hazardous obstacles should be removed from the roadside, and where impossible, barriers and crash cushions should be used [38]. In 1988, Ross presented ways to use field test results and numerical tests based on those tests to assess the technical parameters of barriers, their terminals and crash cushions [39]. In the late 1980s, the European standard EN 1317 is developed on road restraint systems which was subsequently improved [40,41,42]. In 1990 Proctor and Belcher suggest that the United Kingdom is still not fully convinced that crash cushions make sense and provided data which demonstrated that road safety could be significantly improved owing to the devices [43]. The 1990s, an increase in crash cushion application was seen in the Netherlands among others [44]. The period also marks the development of computer-based expert systems that support designers in their choice of restraint systems, including barrier terminals and crash cushions [45]. The work of Ries et al. presents the results of analyses of vehicle impact speeds into concrete barrier terminals and the consequences of such impacts [46]. In 1993, NCHRP 350 report was developed that normalised test procedures for road restraint systems [47]. Work is ongoing on new designs of crashworthy terminals such as the crashworthy terminal for box-beam and W-beam guardrails [48,49,50]. In his research, Elvik looks at how crash cushions and crashworthy terminals reduce accident severity [51]. Numerical tests of crashworthy terminals and crash cushions are developed [52,53]. Systems are built for terminal and crash accident data management [54,55]. In the new millennium, improvements are made to road-restraint systems making them more technologically advanced. In the USA, new guidelines are developed for road restraint systems called MASH [56] and followed when conducting crash tests of crash cushions [57,58]. The scope of numerical tests is extended. The tests are used as tools for verifying new solutions [4,59,60,61]. Authors work on improving numerical models ensuring that they best represent real test results. As an example in [62,63], the authors focussed on analysing cable barriers, works [64,65,66] covered steel guardrail barriers and [67,68,69] studied concrete safety barriers. In addition, innovative devices are tested that are partly built of plastics [70,71]. As well as barriers, numerical analyses are also used to study other road-safety devices, including terminals and crash cushions [72,73,74,75,76,77]. New technologies and research tools help to verify the effectiveness of the available devices [78,79,80,81,82] and confirm the effectiveness of road restraint systems [83]. Bligh and Mak point out that crash terminals should be tested for impacts into the most critical points [84]. Work zones are now protected using trailer-attenuating cushions [85,86,87]. Roper et al. states that the devices used in Australia, including terminal ends, should be replaced with safer solutions [88]. Crash terminals are improved [89,90,91], as well as cable barriers [92,93], concrete barriers [94], and crash cushions [95,96]. More economically efficient crash cushions are sought for use on roads with varying traffic volumes and traffic composition [97,98,99,100]. Effectiveness is also tested for device life cycle [101]. Crash cushions are becoming more and more prevalent [102]. Barrios et al. identifies the hazards to motorcyclists caused by safety barriers including barrier terminals and crash cushions [103]. Roque and Cardoso presented analysis results for impact severity levels on roads across Europe, including terminals and crash cushions and indicated that safer solutions are required [104]. There was a similar study in the USA by Johnson and Gabler [105]. Analyses of the effects of barriers and crash cushions on the level of road safety should also include the fact that the devices may affect sight distance [106]. Crash cushions at entrances to road tunnels were studied by Kunc et al. [107]. Other research points out that crash cushions are required to protect fixed roadside objects [108]. The work by Carrigan and Ray presents analyses of the frequency of specific types of crashworthy terminal impacts and compares real data with crash test data [109]. Ray and Carrigan applied meta-analysis to compare the consequences of crashworthy terminal collisions [110]. Other studies have assessed the effects of the distance between the terminal and traffic lane [111] and how different barrier terminals affect the safety of vehicle occupants [112]. Research by Meng et al. using numerical tests shows that damaged crashworthy terminals have an effect on accident consequences [113].

## 2. Materials and Methods

### 2.1. General Characteristics of Crash Cushions and Terminals

**Figure 1:**
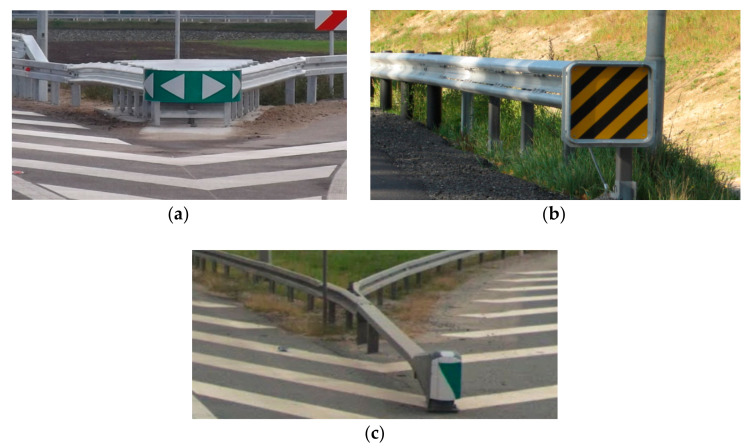
(**a**) Crash cushion, (**b**) crashworthy terminal, (**c**) hybrid device.

Energy absorbing devices are a group of road safety devices designed to absorb the energy of an impacting vehicle and, as a result, to reduce accident consequences which would occur if vehicles hit obstacles directly with no such protection. The main purpose of the devices is to change a potentially serious accident (involving fatalities or serious injuries) into a collision or a slight injury accident. Energy absorbers can be divided into:

- Crash cushions (Figure 1a),
- Crashworthy terminals (Figure 1b),
- Energy-intensive hybrid devices (Figure 1c).

Because the study conducted in this article covers a device which is certified for the European market, the standard EN 1317 [42] will be the document of reference.

The term crash cushion includes all devices that have successfully passed crash tests in accordance with the standard EN-1317-3 irrespective of the type of the material used and type of energy absorption. There are two types of crash cushions: redirective and non-redirective. A non-redirective cushion is designed to slow and contain an impacting vehicle. Its function is to take over the force whose direction is consistent or similar to its construction axis. It is installed in places where head-on vehicle collisions are highly likely as opposed to big angle or side collisions (such as in the case of motorway toll booths). In the case of impacts along the side a redirective cushion works similarly to a safety barrier. Its role is to reduce speed and kinetic energy of an impacting vehicle, change its direction and move it back to the expected direction of travel (cushions placed at the ‘nose’ of an interchange end-terminal). 

Crash and energy-absorbing terminals are road safety devices whose function is to reduce the severity of impact when cars crash into the start or end section of a road safety barrier. They are one of the possible solutions for terminating barriers. There are many types of end-terminals to cater to the needs of different locations. The most common end-terminals include:

- Embedded in the counterscarp—do not absorb the kinetic energy of an impacting vehicle, deflected from barrier axis,
- Anchored in the ground—do not absorb the kinetic energy of an impacting vehicle,
- Absorb the kinetic energy of an impacting vehicle.

Some barrier terminals are unacceptable due to the hazard they pose to car occupants when the car hits the start or end of a barrier; in this case the barrier ends at guardrail level. Moreover, some of the ends that are turned down into the soil behave as launching ramps for impacting vehicles.

Crash terminals can be redirective or non-redirective, single-sided or double-sided and one way or two ways. While the principle of operation is similar as for crash cushions, the main difference is that the terminal must be connected to the safety barrier and the cushion can be independent and work without the barrier.

Hybrid energy absorbers are a type of road safety devices designed to reduce the severity of a crash when cars hit the start or end of a safety barrier and to provide certain functional features of crash cushions. Crash tests are required to confirm the performance expected of both a crash cushion and terminal. 

To answer the research question (Do SafeEnd devices meet the requirements of a hybrid device?), SE device characteristics are presented as well as a list of crash tests, their results and the methodology and results of numerical tests. 

### 2.2. SafeEnd Characteristics 

**Figure 2:**
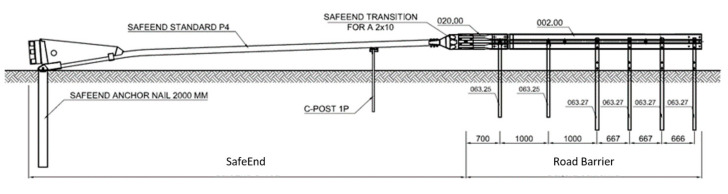
SE geometric diagram and dimensions [mm].

**Figure 3:**
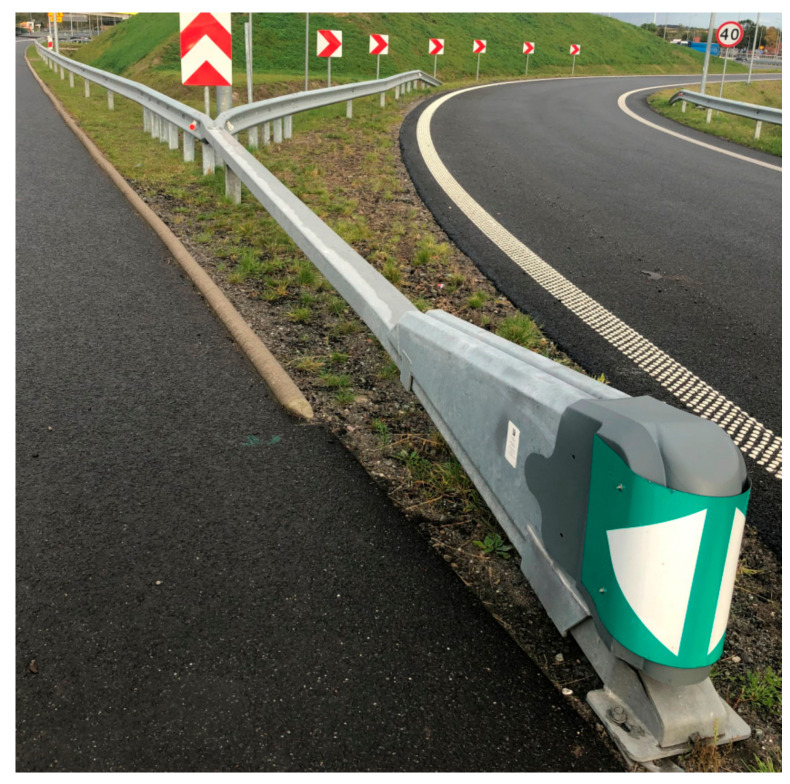
Example of SE application.

SE devices are made of steel elements and are corrosion-protected in a hot-dip galvanising process in accordance with the standard PN EN ISO 1461 [114]. The end part of the SE consists of: ‘backbone’ beam with a rectangular section with its front part placed almost at ground level and rising towards the ending; anchoring and support structure for the device; and a closed movable steel crash box (bumper) with a deformable frontal section (Figure 2 and Figure 3) whose role is to absorb the energy of a frontally impacting vehicle. The bumper is connected to the beam with a brake tensioning belt which goes through a set of brake cylinders in the lowest part of the beam. 

SE devices are installed at the end of steel, concrete and cable barriers acting as crashworthy terminals or energy absorbing cushions U-15a. They are equipped with an amortising element (bumper) which absorbs the energy of the impact. When hit by a vehicle, the bumper deforms and is guided along the upper rail which runs in the direction of travel. Attached to the bumper is a brake tensioning belt. Upon impact the belt is pulled above the rolls together with the box and the entire absorption system is activated and gradually slows down as the vehicle motion resistance grows. As a result, the energy generated in the collision is absorbed and the vehicle is contained on a line defined by the length of the beam within the boundary level of overloads.

While SE devices may provide anchorage for safety barriers (fixed and temporary), when they take over the energy of an impacting vehicle (except side crashes into the transition) they act as a fully self-supporting structure and do not pass the forces onto the barrier. Depending on how safety barriers are installed, they may be used as double-sided, right-hand or left-hand barriers and redirective.

The SafeEnd is the start and end of safety barrier systems designed to absorb the kinetic energy generated by an impacting vehicle. Unlike barrier terminals that terminate in the ground, the SE keeps vehicles safer from being catapulted and rolling over. The SE can be used on single and dual carriageways, both on hard shoulders and central reservations where vehicles may hit the front of the barrier or where vehicles are known to collide frequently with barrier start sections. These devices cannot be used in locations where reverse-direction traffic may exist unless fully tested and evaluated in such a manner.

### 2.3. Research Methods 

Road restraint system research is a complex process and one which requires diverse methods. These include statistical and mechanistic methods [115].

#### 2.3.1. Statistical Methods 

Statistical methods use available detailed data about dangerous road incidents to extract real cases of vehicles hitting safety barriers. Data are collected about the location of the incident, barrier design conditions, road traffic and other circumstances of the incidents. Next, using the data, mathematical models are built which define the relation between barrier functional parameters and the frequency and scale of vehicle crashes into barriers and of other significant factors [14,116,117].

Using the results of tests conducted under the LifeRoSE project [118], a detailed inventory was made of crash cushions and barrier end-terminals on selected sections of national roads. Table 1 presents the inventory.

Two indicators were used to compare the number of devices on sections of the different road classes: crash cushion density WOE and barrier terminal density WZB. This is the number of the devices per 100 km of a specific road class. Analysis of the results shows that per 100 km of road:

- On the network of national roads there are on average about 6 crash cushions and more than 90 barrier terminals,
- On the network of motorways there is on average about 1 crash cushion and more than 106 barrier terminals,
- On the network of express roads there are more than 10 crash cushions and more than 73 barrier terminals.

#### 2.3.2. Mechanistic Methods

##### Field Tests 

**Figure 4:**
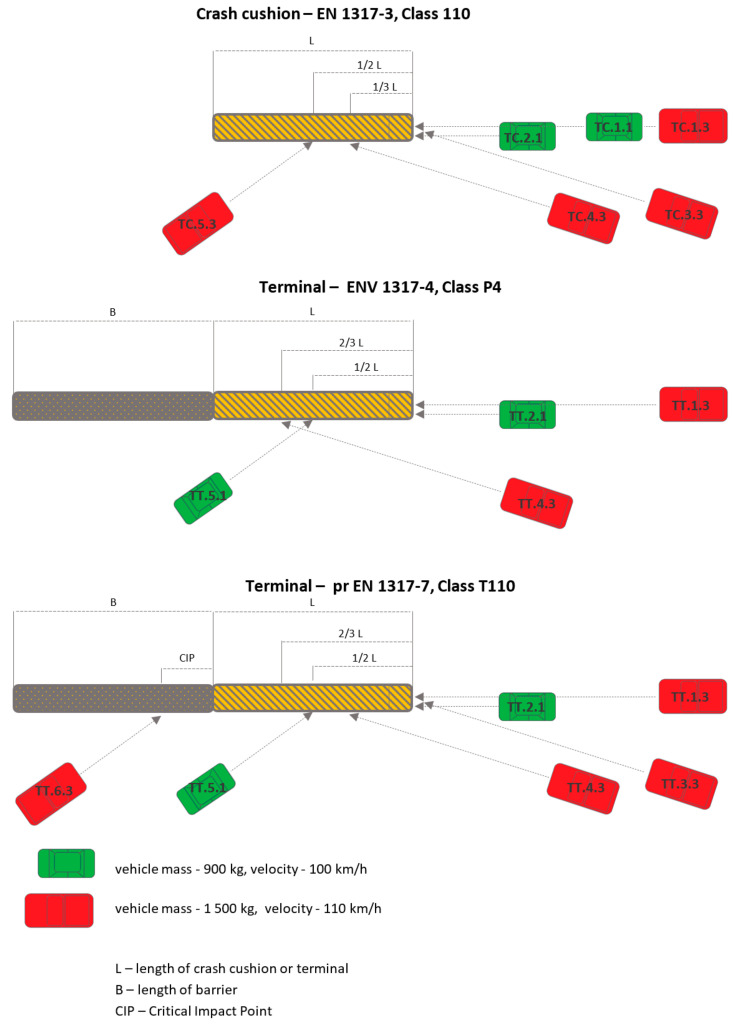
Crash tests for crash cushions and terminals according to standards EN 1317-3, ENV 1317-4, pr EN 1317-7.

The standard EN 1317-3 [119] defines 6 crash tests for crash cushions of the highest performance class (Figure 4):

- Vehicle mass 900 kg, impact velocity 100 km/h: frontal impact at 0° (Test TC 1.1.100), frontal impact at 0° and ¼ vehicle offset (Test TC 2.1.100),
- Vehicle mass 1500 kg, impact velocity 110 km/h: frontal impact at 0° (Test TC 1.3.110), frontal impact at 15° (Test TC 3.3.110), side impact at 15° (Test TC 4.3.110), side impact at 165° (Test TC 5.3.110).

Two documents describe the rules for testing terminals. The first is ENV 1317-4 [120], that defines 4 crash tests for terminals of the highest performance class (Figure 4):

- Vehicle mass 900 kg, impact velocity 100 km/h: frontal impact at 0° and ¼ vehicle offset (test TT 2.1.100), side impact at 165° (test TT 5.1.110);
- Vehicle mass 1500 kg, impact velocity 110 km/h: frontal impact at 0° (Test TT 1.3.110), side impact at 15° (Test TT 4.3.110).

The second document is pr EN 1317-7 [121] (to replace ENV 1317-4), that defines 6 crash tests for terminals of the highest performance class (Figure 4):

- Vehicle mass 900 kg, impact velocity 100 km/h: frontal impact at 0° and ¼ vehicle offset (Test TT 2.1.100), side impact at 165° (Test TT 5.1.110),
- Vehicle mass 1500 kg, impact velocity 110 km/h: frontal impact at 0° (Test TT 1.3.110), frontal impact at 15° (Test TT 3.3.110), side impact at 15° (Test TT 4.3.110), side impact at 165° in combination with road safety barrier (Test TT 6.3.110).

Crash tests conducted in compliance with the standard prEN-1317 part 7, which is the more recent, more advanced and more rigorous test method for crashworthy terminals and transitions, confirm that the device operates properly and meets the criteria of the more accurate and more rigorous test procedure. The assessment method complies with prEN 1317-7 and ensures that the crash cushion was tested for all approaches by a test vehicle as required under EN 1317 part 3, i.e., 5 crash tests:

- Frontal approach, central impact at 0°,
- Frontal approach, impact at ¼ vehicle offset,
- Frontal approach, central impact at 15°,
- Side approach and impact at 15°,
- Side approach and impact at 165°,

nd in addition (not included in part 3 of EN 1317) a sixth crash test, i.e., a side impact at 165° into the transition between the crash cushion and road safety barrier.

The SE device was tested for 8 of 8 vehicle approaches to the crash cushion as defined in EN 1317 for the highest performance classes of crash cushions (Table 2). Traditional energy absorbers (crash cushions, crashworthy terminals) are mostly tested in 4 or 6 vehicle approaches to the device.

The results of SE field tests are given in Section 3.2.

##### Numerical Tests 

Test methodology. Numerical simulations are applied in many fields of engineering. They are tools which help to reduce the design costs because they keep prototyping to a necessary minimum. The design process happens in a virtual space which allows iterative improvements. To ensure the accuracy of a numerical model, a validation process must be conducted. This involves, among others, an objective comparison between real and virtual test results.

The finite element analyses (FEAs) were conducted using the LS-DYNA system (Livermore Software Technology Corporation) [122]. Today it is the most widely used software for numerical crash tests. To integrate the equations of motion, the LS-DYNA uses special form of explicit central difference method called the summed form. This algorithm works well especially for analysing strongly non-linear and rapidly changing phenomena that can comprise extended geometries and variable points of contact. Numerical simulations provide reliable tests of different configurations of vehicles impacting road safety devices and at the same time give good insight into the mechanics of the phenomena involved in the impact. 

For the purposes of this work, the energy absorbing device SafeEnd was tested using selected numerical tests. The following activities were carried out:

- A numerical model of the SafeEnd energy absorbing device was developed,
- The results from a numerical simulation were validated against the results of an actual crash test,
- A parametric analysis was carried out of different impacts with the following variables: speed of impact, angle of impact, place of impact, configuration of the crash cushion-safety barrier relation.

Computational model. The crash test’s numerical model consists of the following elements: SafeEnd device, safety barrier, passenger car, soil and area over which the car moves. The model was designed to analyse vehicular impact into the side of the crash cushion, into the transition between the crash cushion and safety barrier and into a spot along the safety barrier.

**Figure 5:**
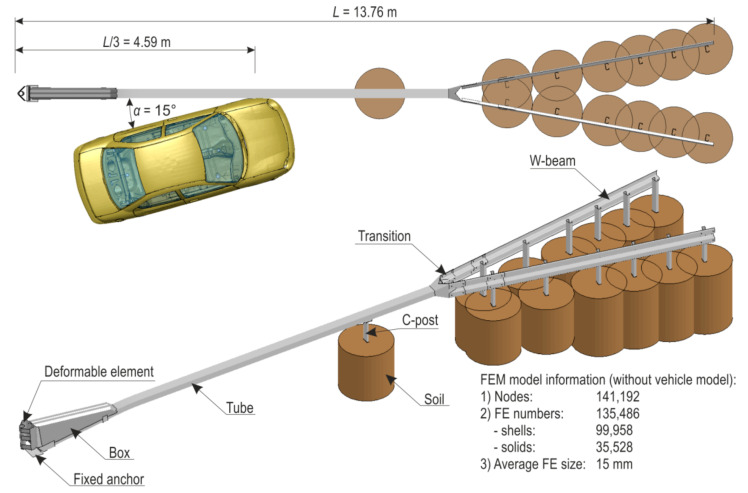
Computational model of the SafeEnd—safety barrier—vehicle system.

The main parts of SafeEnd’s and the safety barrier’s computational model include the following: anchors, rectangular tube, steel box, C-post supporting the beam, transition between SafeEnd and safety barrier, posts and safety barrier profiles. The parts were modelled using shell finite elements (FEs). The average length of the side of a FE is app. 15 mm. The steel parts of road safety devices were assigned an elasto-visco-plastic material model with the strain-rate effects in accordance with the Cowper-Symonds model. That model also accounts for the effects of a damage prior to an element erosion. To discretise bolts, solid FEs were used with assigned damage criteria due to maximum forces and plastic strains. The device’s computational model was supplemented with a soil model in the form of a cylinder under each post. The soil was discretised with solid FEs. This is a common approach and relevant examples can be found in the literature, e.g., [66,123]. The car’s travel area is rigid and was modelled using shell FEs. The rectangular tube that terminates in the ground was constrained by fixing all degrees of freedom. This reflects the actual mounting of the device using a stiff tube anchor which transfers a large part of the impact energy and helps to disperse it in the ground. The discretisation of the SafeEnd device includes 141,192 nodes comprising 135,486 finite elements, of which 99,958 are shells and 35,528 are solids. Figure 5 shows the computational model setup.

Numerical simulations were conducted using the numerical model of a Dodge Neon vehicle whose weight is 1500 kg. The model is commonly used in numerical analyses (see e.g., [66]) and is generally available from on-line resources (*open source*). To improve its numerical stability, the vehicle was modified. The vehicle’s discretisation consists of 284,837 nodes and 271,614 finite elements, including 268,687 shells and 2853 solids. 

The computations take account of the mass of the model parts and mass damping of the system. This helps to reduce non-physical vibrations of high frequencies which are characteristic of computations utilizing explicit central difference method. To simulate contact between the vehicle- road safety device-soil-road system, advanced algorithms are used which symmetrise contact using the penalty-based method.

Model validation. A simulation of a crash test of a SafeEnd energy absorbing device was conducted. The impact conditions are consistent with the test TC 4.3.110 in accordance with the standard PN-EN 1317-3:2010. They are as follows: velocity of impact 110 km/h, angle of impact 15°, place of impact at 1/3 of system length, i.e., 4.59 m from the beginning of the device (see Figure 5). Simulation results were compared with real crash test results.

**Figure 6:**
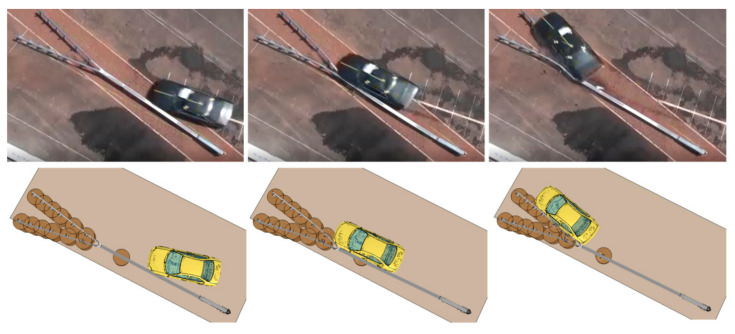
Comparison of the real test (**top**) with the numerical simulation (**bottom**), top down view.

**Figure 7:**
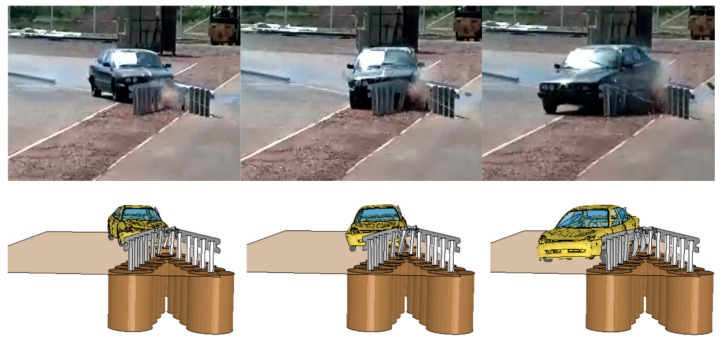
Comparison of the real test (**top**) with the numerical simulation (**bottom**), front view.

**Figure 8:**
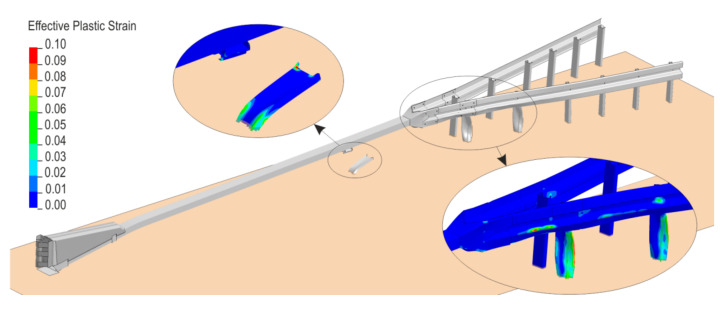
Deformation of the system.

In the numerical test after the vehicle had hit the crash cushion, it was contained and effectively redirected. Following the impact, the rectangular tube came off its support C-post and this post was bent down to the ground. Next, the vehicle hit the first two posts after the transition, bent and twisted them. The w-beam was deformed. Figure 6 and Figure 7 present a comparison between the numerical simulation and the real crash test. Figure 8 shows the deformation of the safety device obtained from the numerical simulation. In the Figure 6, Figure 7 and Figure 8 the soil in the form of the cylinders is visible. In the simulation the acceleration severity index ASI, which is a dimensionless index for defining the effect of the impact on car occupants, is 1.0 (0.96) (ASI = 1.0 in the real test). The theoretical head impact velocity THIV is 23 km/h (and THIV = 20 km/h in the real test). The vehicle comes into contact with the device along a section of 7.3 m (7.6 m in the real test). 

**Figure 9:**
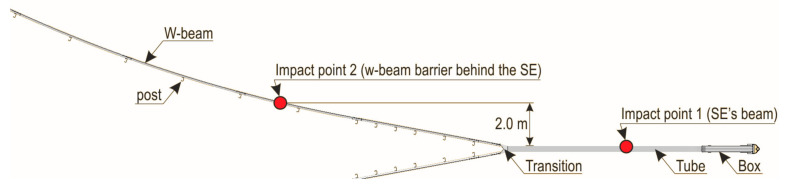
Setup of parametric analysis.

Setup of parametric analysis. After validating the numerical model, a parametric analysis was performed. For the parametric analyses, the model was adjusted, i.e., the safety barrier section was extended. In the model the barrier is installed on a horizontal curve. The discretisation of the numerical model for parametric analyses which consists of SafeEnd devices, the w-beam barrier, vehicle and the vehicle’s travel surface, consists of 589,568 nodes and 563,528 Fes. The simulations carried out were divided in two groups: impact into the SE device in the place as in the field test (Impact point 1), and impact into the road safety barrier on the horizontal curve of the road, in the place which results from the intersection of the barrier with a line parallel to the SE and distant from the SE device by 2.0 m (Impact point 2). The setup is presented in Figure 9. Within those two groups, different speeds and angles of the approach were tested. Additionally, the different radii of the arcs of the barrier connected to the system were also considered. The simulation results are presented in detail in Section 3.3 and discussed in Section 4.

## 3. Results

### 3.1. Statistical Analyses 

**Figure 10:**
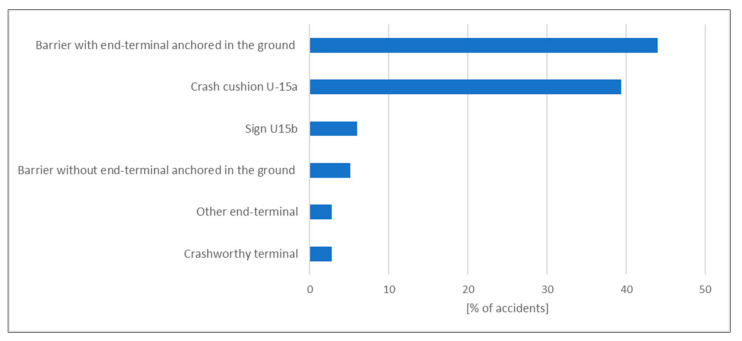
Share of incidents involving safety barrier terminals [68].

The LifeRoSE database for 2017 includes nearly 220 incidents involving damage to barrier terminals and crash cushions. The highest number was recorded for barrier terminals ending in the ground (44.0% of all incidents) and crash cushions (39.4% of all incidents). Incidents involving other barrier terminals were the lowest at 6 incidents representing 2.8% of all incidents (Figure 10).

There were 85 incidents involving energy absorbing devices, of which 79 were classified as collisions (93%) and 6 as road crashes (7%). More than 80% of crash cushion incidents involved frontal vehicular impacts. 

Out of all LifeRoSE incidents, 6 involved hitting an SE (all of them were collisions). All cases of damaged SE devices were caused by frontal impacts of vehicles. In each of the recorded cases, SEs responded properly meaning that they reduced the consequences of vehicles crashing into an unsecured roadside object or obstacle. 

**Figure 11:**
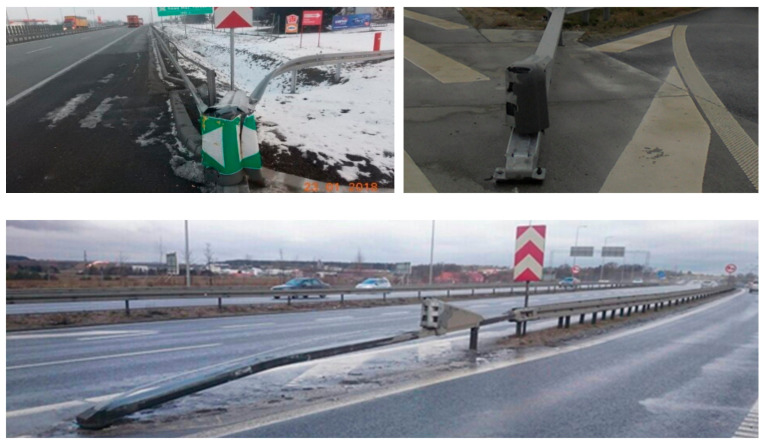
Condition of SE devices after vehicle collisions (source: GDDKiA).

The LifeRoSE project used a database of incidents involving road restraint systems which was made available by the General Directorate for National Roads and Motorways (the GDDKiA). Figure 11 presents examples of the consequences of vehicle collisions with SE devices.

### 3.2. Field Tests 

The SafeEnd device was tested to the standard prEN 1317 part 4 and the results demonstrate its proper performance as a crash cushion. 

**Figure 12:**
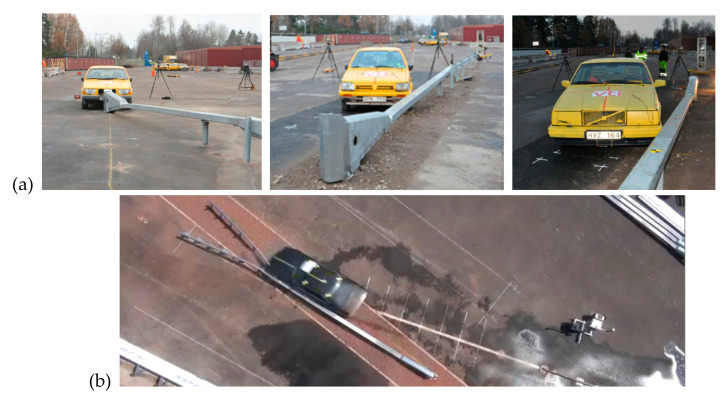
Field tests (**a**) Sweden, (**b**) Poland.

When the device was first tested in 2015 in Sweden (by the VTI research institute) to the standard prEN 1317- part 4 and 7, in all cases the results were positive, including the ASI parameter with the acceleration severity index at level B. The impact it refers to gives the worst ASI result, i.e., a side impact at 165° with a 900 kg vehicle (TT5.1.100) (Figure 12a). The test is carried out solely in a test procedure defined for safety barrier transitions and crash terminals. Tests of crash cushions include an analogous test, but with a 1500 kg vehicle (TC5.3.100). For all other tests the severity impact was in class A.

The device was subsequently tested in 2020 in Poland (Research Institute of Roads and Bridges) to the harmonised most recent version of the standard EN 1317-3:2010 (Figure 12b). A set of tests was performed for the highest speed class of 110 km/h as set out in the standard. In all cases the results were positive including the ASI, i.e., impact severity at level A. This demonstrated that the SafeEnd energy absorbing device met all requirements defined in the standard PN-EN 1317-3 for the highest class of the reference speed.

The tests have helped to solve the research problem and demonstrated that SE devices can be used as hybrid energy absorbers which meet the role of both a safety barrier terminal and a crash cushion placed before a barrier. 

**Figure 13:**
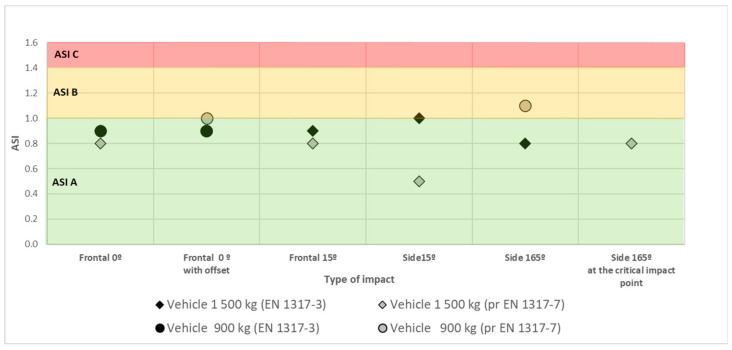
Results of ASI tests from SE field crash tests.

Table 3 and Figure 13 show a cumulative list of the crash test results for a 900 kg vehicle and a 1500 kg vehicle.

### 3.3. Numerical Tests 

**Figure 14:**
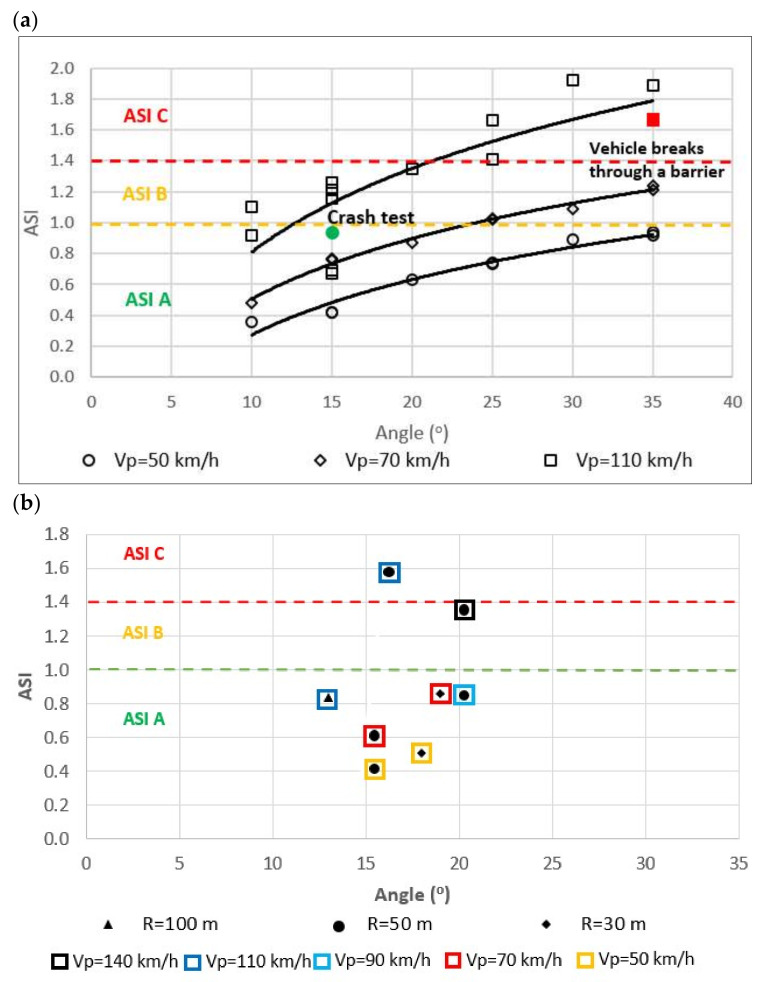
ASI rates achieved in numerical simulations of a vehicle impacting: (**a**) the SE’s beam, (**b**) a safety barrier behind an SE.

**Figure 15:**
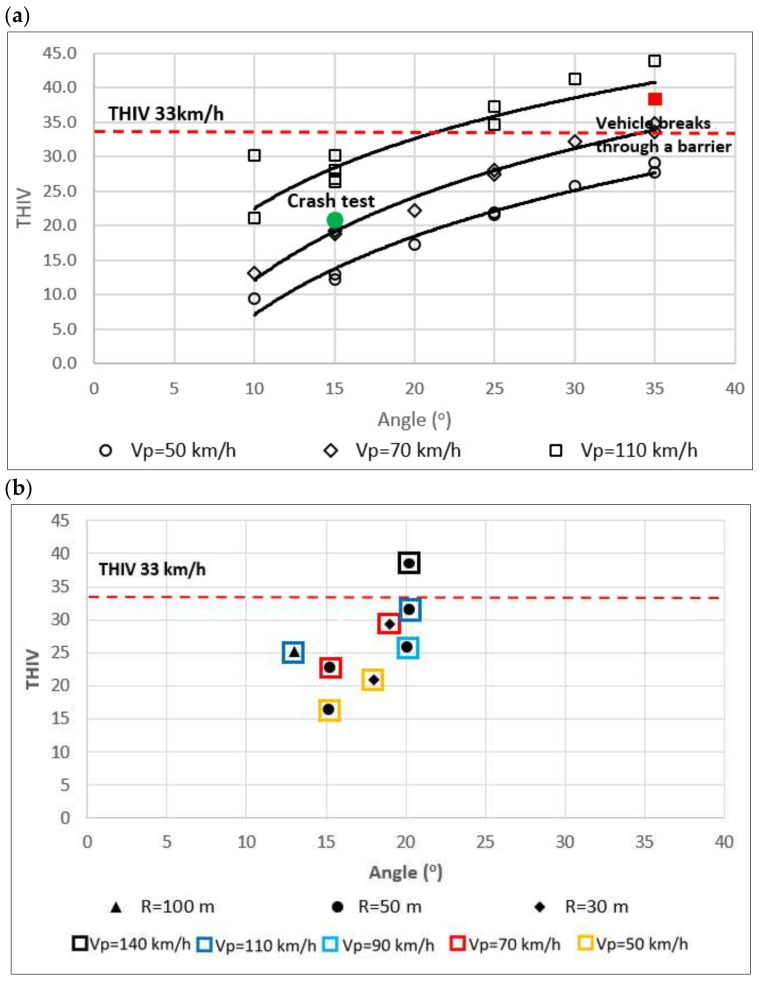
THIV rates achieved in numerical simulations of a vehicle impacting: (**a**) the SE’s beam, (**b**) a safety barrier behind an SE.

To confirm the results of field tests, numerical tests were analysed. The results of numerical crash tests for the SE device were positive for ASI and THIV (Figure 14 and Figure 15):

- Vehicle impacting (vehicle mass 1500 kg) an SE device at different angles KAT (10°–35°) and at different impact speeds Vp (50 km/h, 70 km/h, 110 km/h),
- Vehicle impacting a section of the barrier after the transition to the SE device at KAT = 10° and impact speed Vp (50 km/h, 70 km/h, 110 km/h),
- Vehicle impacting laterally the barrier after the SE device at different angles KAT (10°–35°) and at different impact speeds Vp (50 km/h, 70 km/h, 110 km/h) and on ramps with a curve radius R (30 m; 50 m; 100 m).

## 4. Discussion

The answer to the research question ‘Can SE devices be used as hybrid devices fulfilling the role of both an end-terminal, barrier terminal and crash cushion placed before an obstacle?’ is affirmative. The arguments behind this claim are presented below.

The SafeEnd energy absorber successfully passed field crash tests that are required of crashworthy terminals (according to prEN 1317-4 and 7). The SafeEnd energy absorber successfully passed field crash tests that are required of crash cushions (according to PN-EN 1317-3).

In addition, the results of SafeEnd U-15a numerical crash tests were also positive. 

In the case of a side impact into the SafeEnd’s beam, the simulation tests involved a vehicular impact (1500 kg vehicle mass) into the device at different angles KAT (10°–35°) and for different impact velocities Vp (50 km/h, 70 km/h, 110 km/h). The simulations established that:

- SE guardrail is not penetrated for velocity Vp = 110 km/h and angle not greater than 30°, and in the case of impact velocity below 110 km/h and angle up to 35° (Figure 15),
- The acceptable level of impact severity is (measured with ASI) for 110 km/h velocity and angle not greater than 25°, and in the case of impact velocity below 110 km/h at angle up to 35°,
- The acceptable level of driver’s head impact velocity THIV for 110 km/h and angle not greater than 25°, and in the case of impacts into the device at velocity below 110 km/h and angle up to 35°.

In the case of vehicle impact into the section of the barrier after the SafeEnd transition, the objective of the simulations was to test vehicle impact into the barrier at angle KAT = 10° and vehicle impact velocity Vp (50 km/h, 70 km/h, 110 km/h). Analysis of the simulations shows that the values of kinetic energy of the vehicle impact into the device and into the transition and impact severity indices are no different, i.e., the stiffness and performance of the transition are similar to that of the SafeEnd guardrail.

**Figure 16:**
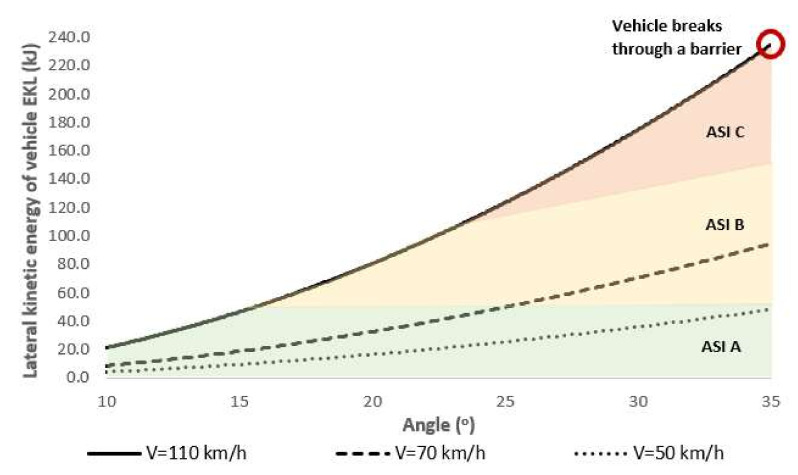
Synthetic results of the effects of side impact angle KAT and velocity of vehicle impact Vp into the SafeEnd U-15a guardrail on lateral kinetic energy of vehicle EKL and impact severity rate ASI.

In the case side vehicle impacts into the barrier after the SafeEnd device, the objective of the simulations was to test vehicular impact into the barrier at different angles KAT (10°–35°) and for different impact velocities Vp (50 km/h, 70 km/h, 110 km/h) and at ramps with a curve radius R (30 m; 50 m; 100 m). The simulations were conducted for the following cases (Figure 16):

- Velocity of errant vehicle Vp = 110 km/h, on a ramp with a radius R = 100 m,
- Velocity of errant vehicle Vp = 70 km/h, on a ramp with a radius R = 50 m,
- Velocity of errant vehicle Vp = 50 km/h, on a ramp with a radius R = 30 m.

**Figure 17:**
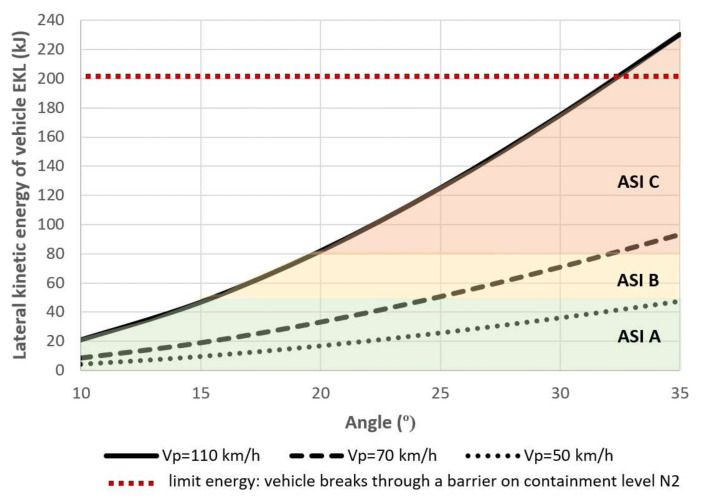
Relations between lateral kinetic energy EKL of a vehicle impacting a barrier placed after a SafeEnd device and vehicle velocity Vp (and related to ramp radius R) and impact angle KAT based on the simulation tests.

Analyses of the simulation tests and tests that the authors conducted under the RoSE research project (RID-3A) [2] established that:

- The safety barrier guardrail is penetrated by a vehicle for energy higher than 200 kJ, such lateral kinetic energy was not achieved in the simulation tests (Figure 17),
- The acceptable impact severity level (measured with ASI) for 110 km/h and angle not greater than 15°, and in the case of impact velocity lower than 110 km/h at 35°.

Considering the test results, the SafeEnd energy absorber should be classified as an energy-absorbing device. Tested to the standard prEN 1317 part 7, the SE device was tested more rigorously than in the case of EN 1317 part 3 or 4. Because the device combines the technical performance and safety level of traditional crash cushions and crashworthy terminals, it is clearly an innovation. Its innovation lies in its ability to perform under specific conditions (e.g., on ramps) as a hybrid device, i.e., a crash cushion or crashworthy terminal and offers the advantages of both. 

## 5. Conclusions

Numerous concerns were raised by General Directorate for National Roads and Motorways staff and auditors regarding SafeEnd crashworthy terminals and crash cushions placed at diverging lanes and ramps of express roads. Most of the objections had to do with SafeEnd used as a hybrid device combing the role of a crashworthy terminal and crash cushion placed before an obstacle and how it is connected with the barrier. 

To clarify the doubts, the results of crash tests and simulation tests were evaluated, and the following conclusions were formulated:

(1) The results of the field tests (crash tests) and numerical tests (simulation tests) and analyses of the results confirm that the SafeEnd U-15a energy absorbing device as an innovative hybrid device can be used as both a crashworthy terminal to protect barrier ends and a crash cushion to protect roadside obstacles under specific conditions.
(2) The lateral kinetic energy of a barrier impacting vehicle and the impact severity level depend on the location of the SafeEnd energy absorbing device relevant to the road edge, emergency lane and at interchange ramps and on the speed of the vehicle and ramp radius (angle of vehicular impact into barrier).

Subsequent research should aim to define the location of crash cushions and hybrid devices on e.g., interchanges from the perspective of road safety.

## Figures and Tables

**Table 1:** Cushions and barrier end-terminals on sections of national roads.

Road Class	Road Length	Number of Crash Cushions	Number of Barrier Terminals	Crash Cushion Density	Barrier Terminal Density
	L	NCC	NBT	DCC	DBT
	km	pcs.	pcs.	pcs./100 km	pcs./100 km
Motorways	880.5	10	934	1.0	106.0
Express Roads	703.9	70	514	10.4	73.0
Main Roads	299.0	15	257	5.0	86.0
Total:	1883.3	107	1705	5.8	90.5

**Table 2:** SE Crash tests.

Test Code	Type of Impact	Mass	Crash Cushions		Terminals			
			PN-EN 1317-3		ENV 1317-4		pr EN 1317-7	
			Required	Delivered	Required	Delivered	Required	Delivered
1.1	Frontal impact 0°	900	x	x				
1.3		1500	x	x	x	x	x	x
2.1	Frontal impact 0°, ¼ vehicle offset	900	x	x	x	x	x	x
3.3	Frontal impact 15°	1500	x	x			x	x
4.3	Side impact 15°	1500	x	x	x	x	x	x
5.1	Side impact 165°	900			x	x	x	x
5.3		1500	x	x				
6.3	Side impact 165° into transition element	1500					x	x

**Table 3:** Resulting test parameters for an SE device.

Standard Conformity	PN-EN 1317-3	prEN 1317-4	prEN 1317-7
Performance Class	110	P4	T 110
Test Velocity	100 km/h, 110 km/h	100 km/h and 110 km/h	100 km/h and 110 km/h
Test Vehicle Mass	900 kg and 1 500 kg	900 kg and 1500 kg	900 kg and 1500 kg
Number of Crash Tests	6	4	6
Impact Severity ASI	A	B	B
Redirection Zone	Z2	Z1	Z3
Lateral Displacement Class	D1	D1.1	S0.5 and T1.0
Durability	According to PN-EN ISO 1461	According to PN-EN ISO 1461	According to PN-EN ISO 1461

## Data Availability

Not applicable.
